# Application of an Artificial Intelligence Trilogy to Accelerate Processing of Suspected Patients With SARS-CoV-2 at a Smart Quarantine Station: Observational Study

**DOI:** 10.2196/19878

**Published:** 2020-10-14

**Authors:** Ping-Yen Liu, Yi-Shan Tsai, Po-Lin Chen, Huey-Pin Tsai, Ling-Wei Hsu, Chi-Shiang Wang, Nan-Yao Lee, Mu-Shiang Huang, Yun-Chiao Wu, Wen-Chien Ko, Yi-Ching Yang, Jung-Hsien Chiang, Meng-Ru Shen

**Affiliations:** 1 Institute of Clinical Medicine College of Medicine National Cheng Kung University Tainan Taiwan; 2 Division of Cardiology, Internal Medicine National Cheng Kung University Hospital, College of Medicine National Cheng Kung University Tainan Taiwan; 3 Department of Clinical Medical Research National Cheng Kung University Hospital, College of Medicine National Cheng Kung University Tainan Taiwan; 4 Department of Medical Imaging National Cheng Kung University Hospital, College of Medicine National Cheng Kung University Tainan Taiwan; 5 Division of Infectious Diseases, Department of Internal Medicine National Cheng Kung University Hospital, College of Medicine National Cheng Kung University Tainan Taiwan; 6 Department of Pathology National Cheng Kung University Hospital, College of Medicine National Cheng Kung University Tainan Taiwan; 7 Department of Medical Laboratory Science and Biotechnology College of Medicine National Cheng Kung University Tainan Taiwan; 8 Institute of Basic Medical Science College of Medicine National Cheng Kung University Tainan Taiwan; 9 Department of Computer Science and Information Engineering College of Electrical Engineering and Computer Science National Cheng Kung University Tainan Taiwan; 10 Department of Family Medicine National Cheng Kung University Hospital, College of Medicine National Cheng Kung University Tainan Taiwan; 11 Department of Obstetrics and Gynecology National Cheng Kung University Hospital, College of Medicine National Cheng Kung University Tainan Taiwan; 12 Department of Pharmacology College of Medicine National Cheng Kung University Tainan Taiwan

**Keywords:** SARS-CoV-2, COVID-19, artificial intelligence, smart device assisted decision making, quarantine station

## Abstract

**Background:**

As the COVID-19 epidemic increases in severity, the burden of quarantine stations outside emergency departments (EDs) at hospitals is increasing daily. To address the high screening workload at quarantine stations, all staff members with medical licenses are required to work shifts in these stations. Therefore, it is necessary to simplify the workflow and decision-making process for physicians and surgeons from all subspecialties.

**Objective:**

The aim of this paper is to demonstrate how the National Cheng Kung University Hospital artificial intelligence (AI) trilogy of diversion to a smart quarantine station, AI-assisted image interpretation, and a built-in clinical decision-making algorithm improves medical care and reduces quarantine processing times.

**Methods:**

This observational study on the emerging COVID-19 pandemic included 643 patients. An “AI trilogy” of diversion to a smart quarantine station, AI-assisted image interpretation, and a built-in clinical decision-making algorithm on a tablet computer was applied to shorten the quarantine survey process and reduce processing time during the COVID-19 pandemic.

**Results:**

The use of the AI trilogy facilitated the processing of suspected cases of COVID-19 with or without symptoms; also, travel, occupation, contact, and clustering histories were obtained with the tablet computer device. A separate AI-mode function that could quickly recognize pulmonary infiltrates on chest x-rays was merged into the smart clinical assisting system (SCAS), and this model was subsequently trained with COVID-19 pneumonia cases from the GitHub open source data set. The detection rates for posteroanterior and anteroposterior chest x-rays were 55/59 (93%) and 5/11 (45%), respectively. The SCAS algorithm was continuously adjusted based on updates to the Taiwan Centers for Disease Control public safety guidelines for faster clinical decision making. Our ex vivo study demonstrated the efficiency of disinfecting the tablet computer surface by wiping it twice with 75% alcohol sanitizer. To further analyze the impact of the AI application in the quarantine station, we subdivided the station group into groups with or without AI. Compared with the conventional ED (n=281), the survey time at the quarantine station (n=1520) was significantly shortened; the median survey time at the ED was 153 minutes (95% CI 108.5-205.0), vs 35 minutes at the quarantine station (95% CI 24-56; *P*<.001). Furthermore, the use of the AI application in the quarantine station reduced the survey time in the quarantine station; the median survey time without AI was 101 minutes (95% CI 40-153), vs 34 minutes (95% CI 24-53) with AI in the quarantine station (*P*<.001).

**Conclusions:**

The AI trilogy improved our medical care workflow by shortening the quarantine survey process and reducing the processing time, which is especially important during an emerging infectious disease epidemic.

## Introduction

In December 2019, a local outbreak of pneumonia was detected in Wuhan, Hubei Province, China. Initially of unknown cause, the pneumonia was quickly determined to be caused by a novel coronavirus [1,2]. The International Committee on the Taxonomy of Viruses termed this novel virus SARS-CoV-2; the disease it causes is called COVID-19 [3,4]. The COVID-19 outbreak subsequently spread to every province of mainland China and then globally, with 1,773,084 confirmed cases and 111,652 deaths as of April 14, 2020 [5].

COVID-19 is considered to be very contagious in human beings [6]. Person-to-person transmission has been documented for COVID-19 [7], and the prevention of in-hospital outbreaks has become an important issue for crowded departments with highly infectious areas, including emergency departments (EDs), outpatient clinics, and inpatient admission wards [8-10]. In response to this, Taiwan implemented a disaster preparation plan by moving to “heightened” military level status, the highest of the three security tiers during peacetime; this is similar to war preparation status, with increased alertness and enhanced caution regarding the possibility of cross-contamination among people, especially within hospitals. As the COVID-19 epidemic situation deteriorates, the burden on quarantine stations outside EDs at hospitals is increasing daily. To address the high screening workload at quarantine stations, all staff members with medical licenses are required to work shifts in these stations. Therefore, it has become necessary to simplify the workflow and decision-making process for physicians and surgeons from all subspecialities.

To address this pandemic, the government of Taiwan reacted with a swift, efficient, and precise response [11]. Taiwan prevented entry to travelers from high-risk regions outside Taiwan as the first step; also, Taiwanese citizens and alien residents were quarantined, screened, and isolated based on specific criteria and conditions announced by the Taiwan Centers for Disease Control (CDC). However, the situation was dynamic and fluid and changed daily, with new CDC announcements requiring immediate protocol changes. To address these criteria in their screening strategy and to reduce the processing time in COVID-19 quarantine stations, National Cheng Kung University Hospital (NCKUH) designed an artificial intelligence (AI) system to address this unmet clinical need. The NCKUH AI team used previous experience from prior smart medicine research and applications to develop a specific computer-assisted technology device to accelerate medical decisions. The goal was to reduce potentially dangerous SARS-CoV-2 exposure by shortening the period of time spent by patients in the quarantine unit. In response to this increasing public health emergency, the NCKUH AI team developed a smart quarantine station for use outside the NCKUH ED to facilitate the screening and survey process. The aim of this study is to demonstrate how the NCKUH AI trilogy of diversion to the smart quarantine station, AI-assisted image interpretation, and a built-in clinical decision-making algorithm improved medical care and reduced quarantine processing times.

## Methods

### The Smart Quarantine Station AI Trilogy

To improve the workflow and efficacy of the COVID-19 infection survey at NCKUH, an AI trilogy policy was employed outside and adjacent to the ED. The three parts of the trilogy were diversion of suspected patients with COVID-19 to the quarantine station; a travel, occupation, contact, and cluster (TOCC) survey on a tablet computer; and an AI-assisted image interpretation feature with clinical strategy decision-making algorithms at the smart quarantine station. Together, these steps facilitated the screening and survey process for suspected cases with or without symptoms and the collection of TOCC histories at the NCKUH.

**Figure 1:**
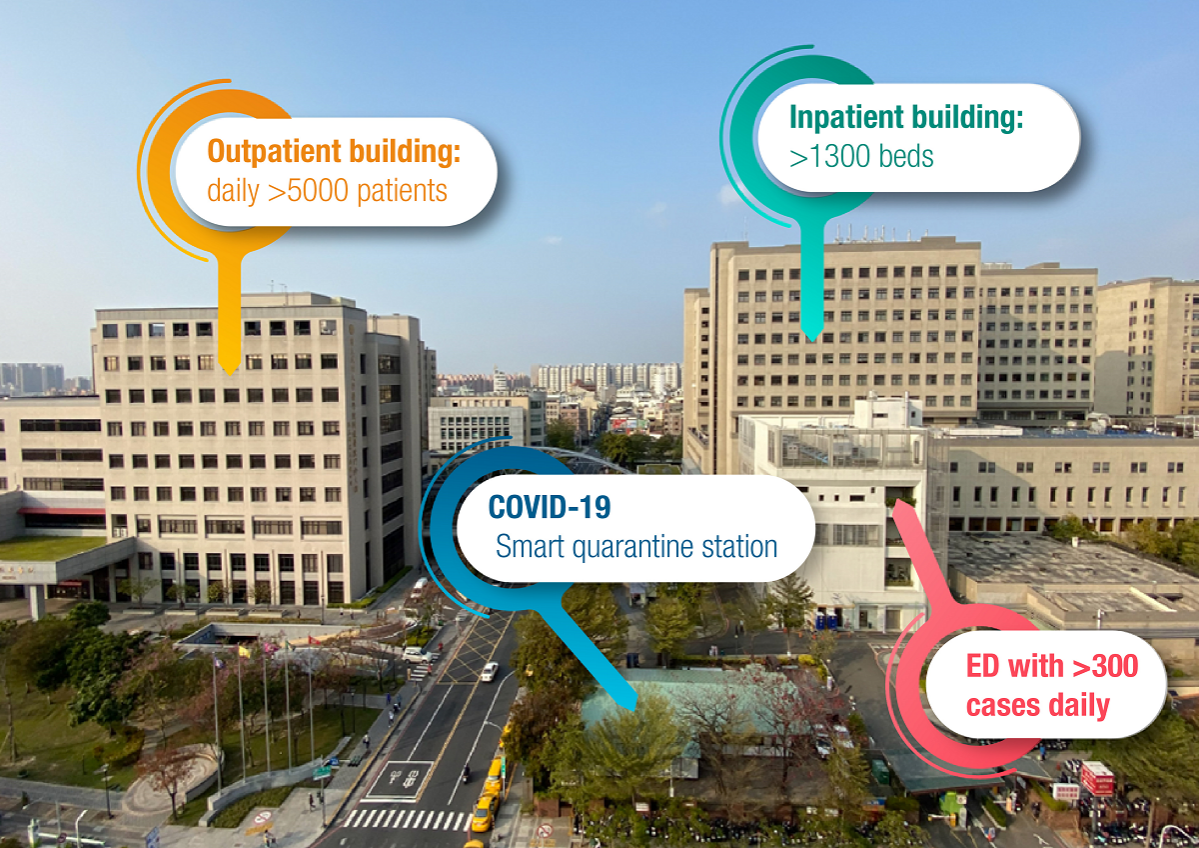
The quarantine station at National Cheng Kung University Hospital. ED: emergency department.

The NCKUH is a referral medical center and teaching hospital with the largest medical service capacity in Tainan, a traditional culture city. Tainan is the sixth largest city in Taiwan, with >1,880,000 citizens. NCKUH has three main care facilities: an outpatient building that houses short-term patient care and clinical laboratories; an inpatient building where long-term care, the intensive care unit, and surgery wards are located; and the ED. With >5000 outpatient visits daily and 1300 inpatient beds in addition to >300 emergency critical care patients in the ED, NCKUH understood the need to prevent the emerging infectious disease epidemic from becoming a local disaster and overburdening the medical system. The lessons learned from Taiwan’s previous experience with the severe acute respiratory syndrome coronavirus (SARS-CoV) outbreak in 2003 have paid substantial dividends during the current COVID-19 outbreak. To address the expected high influx of COVID-19–related hospital visits and prevent in-hospital outbreaks, a quarantine station was established adjacent to the ED. NCKUH immediately set up eight temporary wartime quarantine tents according to the Wuhan Pneumonia Quarantine Action. Simultaneously, a more permanent structured housing facility for long-term quarantine was established near these wartime tents (Figure 1). This effective diversion of patients from the ED to the quarantine tents relieved pressure on the crowded ED. On average, each medical staff member was estimated to examine 35 to 50 quarantine patients per day.

### Workflow Survey and Definition of Survey Time

Because the COVID-19 outbreak was rapidly becoming an epidemic and posed a threat of high risk of exposure to our staff and patients, NCKUH urgently instituted a workflow survey. Patients who visited the quarantine station were asked to complete a questionnaire that included their TOCC history as well as any chronic illnesses, symptoms, or signs. Before they entered any private information, the patients gave signed consent allowing us to use their data and baseline characteristics for subsequent analysis of the workflow. The survey time was defined as the total of the time a patient took to complete the TOCC history with a nurse’s written record and history taking; the time needed to undergo a chest x-ray examination with related inquiry and expert interpretation; and the time to revisit with the examining physician to discuss a medical decision based on the patient workflow recommended by the Taiwan CDC.

The physician would decide whether the patient should be hospitalized, be registered, or recover at home under self-care. Furthermore, the NCKUH staff could access electronic medical records using the tablet computer along with medical AI radiomics of chest x-rays to alert radiologists and pulmonary physicians to screen pneumonia patches earlier than usual. Finally, the embedded clinical decision-making algorithm assisted the staff in assessing potential cases; this could also be useful to physicians who were not familiar with the emerging virus. This process was updated daily using the most recent guidelines from the Taiwan Ministry of Health and Welfare in collaboration with the Taiwan CDC.

### Disinfection Protocol for the TOCC Tablet Computer

To assess if our disinfection process could effectively clean any possible SARS-CoV-2 contamination from a tablet computer surface, we performed an ex vivo study by spraying 20 microliters of a positive SARS-CoV-2 virus solution onto a computer in a P3 laboratory at NCKUH. After the computer surface was air-dried for 30 minutes, it was swabbed by technicians to obtain the first sample (V0), which indicated the surface virus load after initial exposure to the virus. After V0 sample collection, the technicians used 75% alcohol to mimic the disinfection procedure that was recommended in the current Taiwan CDC guideline. Samples V1 and V2 were collected after disinfecting the surface once and twice using 75% alcohol, respectively. The crossing point (CP), which is the maximum second derivative of the amplification curve of the real time–polymerase chain reaction (RT-PCR), was measured to obtain the viral load on the tablet computer surface using the COVID-19 Genesig RT-PCR assay (Primerdesign Ltd) using a LightCycler 480 II PCR platform (Roche Holding AG). A positive viral load was defined by the CP of amplification curve <45 of the RT-PCR result. Two specific genes, RNA-dependent RNA polymerase (RdRp) and Envelope (E) genes, were simultaneously assayed to determine the results.

### AI-Assisted Reading of Chest X-Rays

COVID-19 pneumonia exhibits a milder radiological course than severe acute respiratory syndrome (SARS) and Middle East respiratory syndrome (MERS) pneumonia; only 33% of COVID-19 pneumonia cases in Korea and only 60% of cases in China were reported to have chest x-ray abnormalities [12]. Patients with COVID-19 pneumonia can have negative chest radiographs or computed tomography (CT) images even if their RT-PCR test is positive. According to Pan et al [13], the time course of COVID-19 pneumonia adheres to a clinical progression. The subclinical group has a CT pattern of unilateral and multifocal ground-glass opacities (GGOs) that progress immediately to bilateral and diffuse GGOs. The image may be mixed with light consolidations by the peak of disease severity, which occurs approximately 2 weeks after symptom onset [14]. In current practice, it is difficult to interpret chest radiographs showing GGOs, and this can limit the early diagnosis of COVID-19 pneumonia within the first week of symptom onset. Radiographic deterioration observed in follow-up thoracic imaging implies poor prognosis of COVID-19 pneumonia. Therefore, NCKUH set up portable imaging equipment to accelerate the characterization of chest conditions and limit the transportation of suspected cases to lower the risk of transmission [15].

### Development of AI Models for Chest X-Ray Analysis

**Figure 2:**
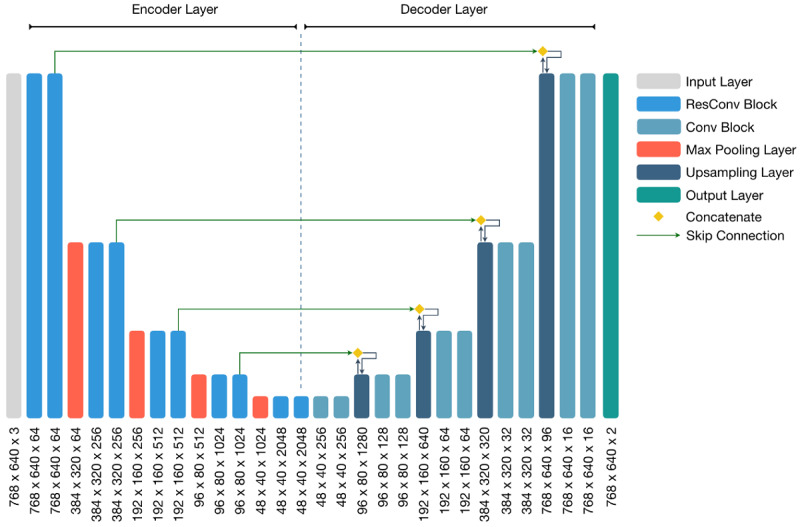
The pneumonia segmentation model based on U-Net. ResNet-50 was used as the encoder backbone model. The squeeze-and-excitation block was added to each ResConv block to enhance feature extraction from feature maps. The skip connection was used to concatenate the encoder feature map with the decoder feature map after upsampling. Conv: convolutional; ResConv: residual convolutional.

The development of AI models for chest x-ray analysis can ease the burden on medical staff and enable more rapid triaging of patients. To detect the precise location of pneumonia sites, we adopted a segmentation model with a class attention map [16]. The pneumonia segmentation model was based on U-Net [17], as shown in Figure 2.

**Figure 3:**
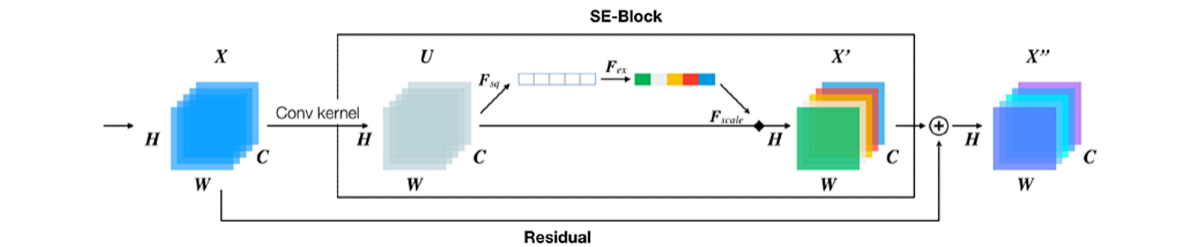
Model of the addition of the SE block to the residual convolutional block. Conv: convolutional; SE: squeeze-and-excitation.

The Conv block was constructed using a 3 × 3 convolutional block and batch normalization [18] and was then activated using a rectified linear unit (ReLU) function. We also adopted a residual network, ResNet-50 [19], with the pretrained weight on the ImageNet as the backbone of this model. Moreover, we leveraged the squeeze-and-excitation block [20], as shown in Figure 3, to capture more significant information in each residual convolutional block.

After *U* was established, the global average pooling in *F_sq_* was used to obtain global information from each feature map. Two fully connected layers were adopted with the ReLU and sigmoid functions to apply a nonlinear transformation to earn attention information in *F_ex_*. The attention weight produced by *F_ex_* was multiplied to feature map *U* to obtain more efficient features of *X’* in *F_scale_*. The input *X* was added to *X’* to obtain the new feature map *X’’* to implement the residual block.

The output masks of this model contained two different predictors: pneumonia and GGO. We used multiple bounding boxes to label all lesions or as many lesions as possible. Because it is possible to observe different illnesses at the same position, we used a sigmoid activation function to leverage two 1 × 1 convolutional kernels, one for detecting pneumonia and one for detecting GGO. Next, we trained this model using pixel-wise binary cross entropy and then minimized the loss function as follows:

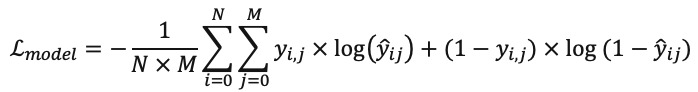



where *N* and M denote the height and width of the input image, respectively, and 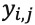 and 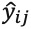 represent the gold standard and predicted value of this pixel, respectively.

### Grouping of Survey Patients

Patients could follow three routes in the NCKUH ED or quarantine station from January 31 to March 17, 2020. Route 1 was the traditional ED route, which did not include AI or the smart device app; this route was followed by patients who visited the ED between 8 PM and 8 AM, when no quarantine station service was available. Route 2 was the quarantine station without AI or the smart device app; most patients followed this route between January 31 and February 5, 2020. Route 3 was the quarantine station with AI and the smart device app, which was mainly followed by patients from February 5 to March 17, 2020. To compare baseline characteristics, we merged Route 2 and Route 3 into a general quarantine station route. Route 1 was recognized as the traditional ED track for screening. Data regarding patients’ preexisting conditions, including coronary artery disease, diabetes, hypertension, chronic obstructive pulmonary disease, underlying malignancy, and chronic kidney disease with or without end-stage renal disease, were all obtained from medical records or the SCAS database. Patients’ symptoms, including dyspnea, cough, stuffed nose, fever, and body temperature, were also recorded individually. Informed patient consent was built into the front page of the tablet computer app before the start of the questionnaire due to the urgent need to collect data while avoiding paper cross-contamination.

### Patient and Public Involvement and Ethical Issues

This study was designed by the investigators. Patients or the public were enrolled in the research study when they visited the quarantine station and agreed to participate before answering the SCAS questionnaire on the tablet computer. The NCKUH Institutional Review Board approved our use of information and images obtained from the quarantine station (A-ER-109-149). Furthermore, due to the pandemic, the dissemination of results to the study participants was not applicable.

### Statistical Analysis

Statistical analyses were performed using SPSS Version 22.0 (IBM Corporation). Continuous data are presented as mean (SD) or median (IQR) depending on the distribution. Dichotomous data are presented as n (%). Comparisons were conducted with nonparametric statistics using the Wilcoxon rank sum test or the Mann-Whitney U test for continuous variables. *P* values <.05 were considered significant.

## Results

### Overview

**Figure 4:**
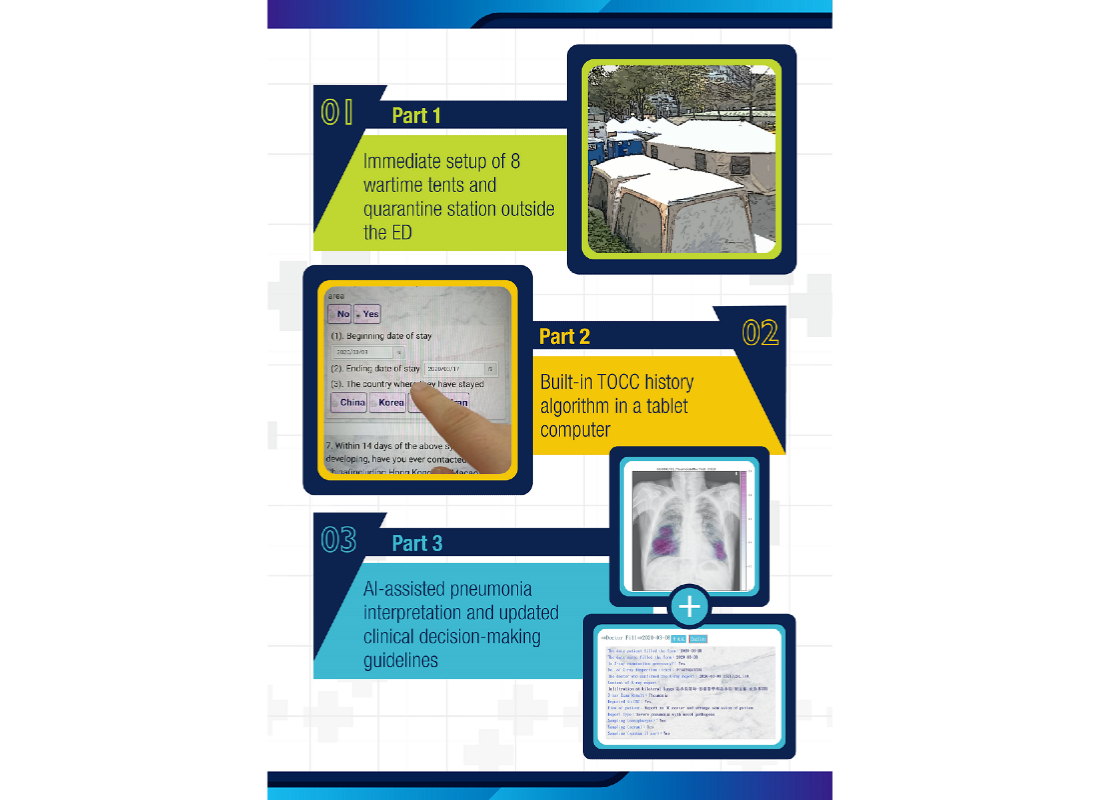
Synergetic combination of quarantine station establishment, smart patient processing, and AI to improve the efficiency and safety of patient processing during the COVID-19 pandemic. ED: emergency department; TOCC: travel, occupation, contact, and cluster.

To improve the efficiency of the survey to determine COVID-19 infection risk and safely reduce crowding in the ED, NCKUH in Taiwan established an AI trilogy comprising three parts. First, we built a quarantine station with a SCAS to accelerate the workflow (Figure 4). SCAS is a clinical decision tree algorithm that integrates the structured format of the TOCC history recording, AI-assisted interpretation of chest x-rays, and the clinically recommended workflow. Patients at the quarantine station who were suspected of having COVID-19 used a tablet computer to answer the SCAS questionnaire on their TOCC history, with adequate alcohol disinfection between users. To avoid cross-infection between front-line medical staff and patients, all physicians and nurses used independent computers.

### Disinfection Process Assessment

**Figure 5:**
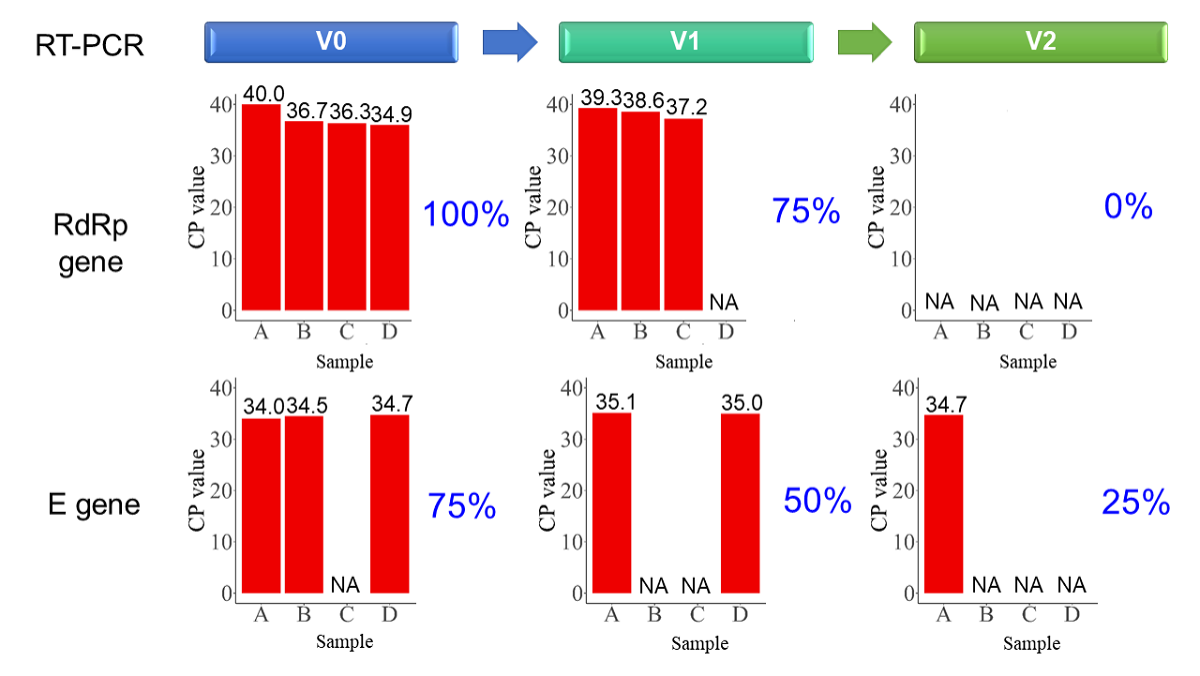
Ex vivo study to determine the efficiency of disinfection of the tablet computer surface. Samples were collected from the tablet surface before disinfection (V0) and after the first and second disinfection processes using 75% alcohol (V1 and V2, respectively). The percentages indicate the positive rate (C*P* value <45 as positive) of the real time–polymerase chain reaction (RT-PCR, N=4 for each experiment). CP: crossing point. RdRp: RNA-dependent RNA polymerase gene. E: Envelope gene.

To assess if our disinfection process of the tablet computer device using 75% alcohol sanitizer was effective and removed all residual viruses from the surface of the device, we performed an ex vivo study. First, 20 microliters of a positive SARS-CoV-2 virus solution was sprayed onto the computer surface. Subsequently, 75% alcohol was used to clean and disinfect the computer surface (Figure 5). We tested four samples without 75% alcohol treated and found that the initial results were 100% positive tested by SARS-CoV-2 RT-PCR. Interestingly, the CP values for RdRp gene were 75% and 0% positive after the first and second disinfection procedures using 75% alcohol on the tablet computer surface, respectively. Similarly, the initial CP value for the COVID-19 E gene was 75% positive, and it decreased to 50% and 25% positive after the first and second alcohol disinfection procedures, respectively. These results supported our proposed tablet computer cleaning protocol of two careful disinfection processes using 75% alcohol between uses of the tablet.

### AI Chest X-Ray Interpretation

**Figure 6:**
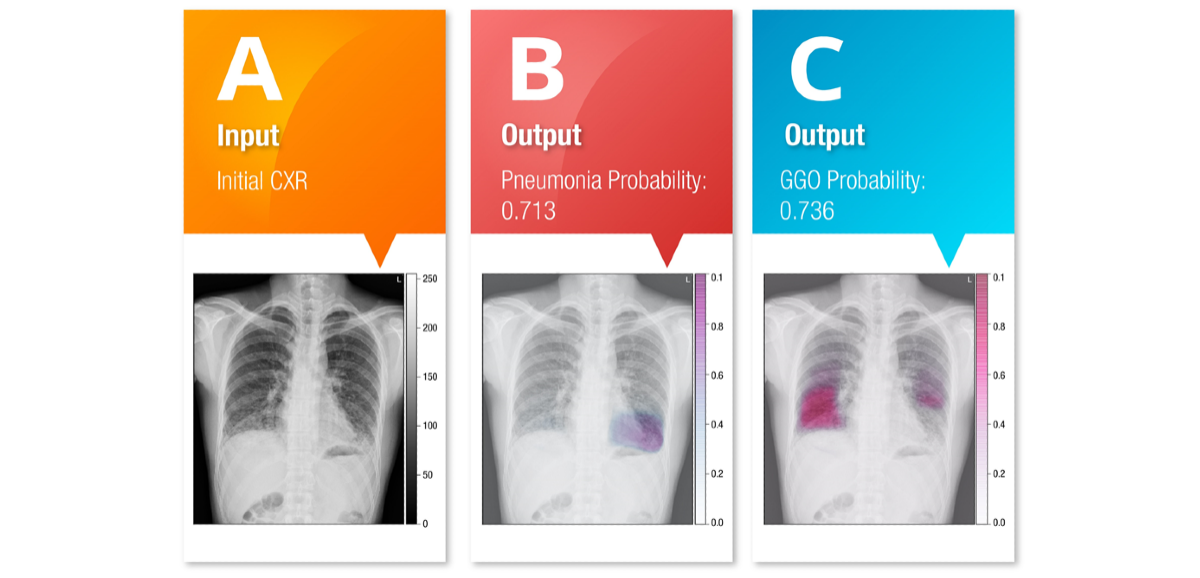
The artificial intelligence (AI) model for pneumonia detection incorporated into the smart clinical assisting system. (A) An original chest x-ray is automatically retrieved from the picture archiving and communication system and then interpreted by the AI model. (B) A consolidative lung is detected, and the diseased site is illustrated using a heat map. (C) A light consolidation GGO is identified by the AI model. CXR: chest x-ray; GGO: ground-glass opacity.

**Figure 7:**
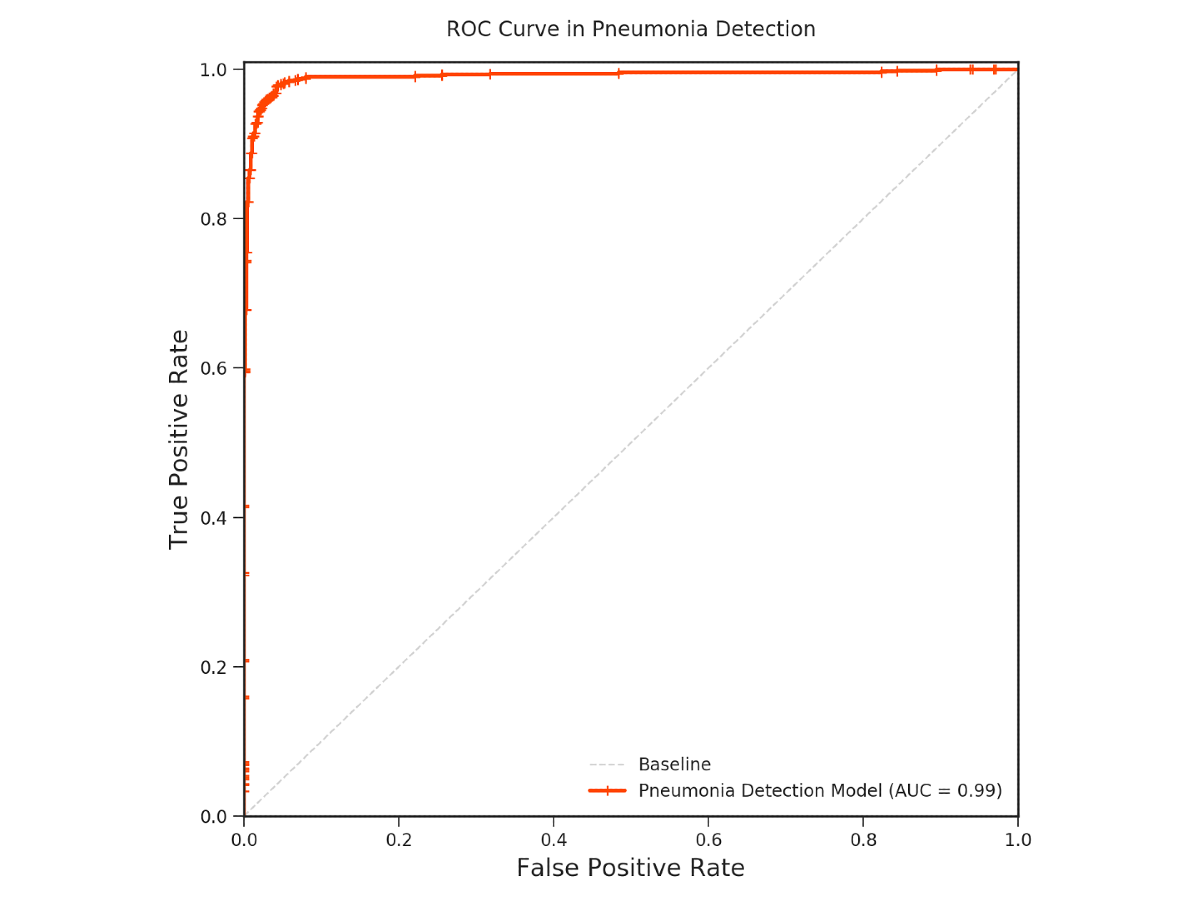
AUC of the AI chest x-ray interpretation system for recognizing pulmonary infiltrates on chest x-rays. AUC: area under the ROC curve; ROC: receiver operating characteristic.

To optimize AI chest x-ray interpretation, we retrospectively retrieved 4000 chest x-rays from our image archiving and communication system and identified 682 posteroanterior chest x-rays with pneumonia and 692 normal chest posteroanterior x-rays. All poor-quality anteroposterior chest radiographs and chest x-rays were excluded. To enhance the detection rate of light consolidations, we selected 46 cases with CT-evidenced GGOs and posteroanterior chest X-rays from the same day as the CT examination to serve as the GGO training data set (Figure 6). Our AI mode for quickly recognizing pulmonary infiltrates on chest x-rays was merged into the SCAS, with an area under the receiver operating characteristic curve (AUC) of 0.99 (Figure 7), sensitivity of 94.1%, specificity of 95.1%, and accuracy of 94.6% using the training data set. Furthermore, we used cases of COVID-19 pneumonia from an open source data set in GitHub [21] including 59 posteroanterior and 11 anteroposterior chest x-rays to test the model. We achieved detection rates of 55/59 (93%) in posteroanterior and 5/11 (46%) in anteroposterior chest x-rays compared to interpretation by one cardiothoracic radiologist with 14 years of experience.

Our AI mode for quickly recognizing pulmonary infiltrates on chest x-rays was merged into the SCAS, with an AUC of 0.99, sensitivity of 94.1%, specificity of 95.1%, and accuracy of 94.6% using the training data set.

### Clinical Data of Study Participants

The screening population of patients who visited the NCKUH ED and who agreed to provide their data for the study (N=643) was divided into two groups: the traditional ED group (n=281) and the smart quarantine station group (n=362). The baseline characteristics of these two groups are compared in Table 1.

The average age of the patients who visited the quarantine station was 35.6 years (SD 13.1), which was similar to the average age (34.7 years, SD 12.3) of patients visiting the traditional ED route. The most dominant trait in the TOCC was the traveling history for both groups, followed by the contact history. The screening population was a lower risk cohort, with low prevalence rates of hypertension (9/281, 3.3%, and 19/362, 4.4%, in the ED and quarantine station groups, respectively) and chronic kidney disorder (8/281, 2.8%, and 5/362, 1.5%, in the ED and quarantine station groups, respectively); this suggests similar chronic illness histories in the two groups. Most patient symptoms manifested as a cough at the initial presentation (ED vs quarantine station: 140/281, 49.8% vs 226/362, 62.4%; *P*=.31), followed by a stuffed nose (ED vs quarantine station: 88/281, 31.3%, vs 166/362, 45.8%; *P*=.31) and fever (ED vs quarantine station: 78/281, 27.7%, vs 134/362, 37%; *P*=.37). In general, the comparison between the two groups showed no difference in the distributions with respect to baseline age, gender, pre-existing conditions, or symptoms.

**Table 1:** Comparison of clinical data of patients seeking treatment in the traditional ED and at the smart quarantine station during the COVID-19 epidemic from January 31 to March 17, 2020 (N=643).

Characteristic		ED^a^ (n=281)	Smart quarantine station (n=362)	*P* value
Age (years), mean (SD)		34.7 (12.3)	35.6 (13.1)	.41
Sex (male), n (%)		144 (51.2)	165 (45.6)	.11
**TOCC^b^ history, n (%)**				
	Traveling	10 (62.4)	308 (49.0)	.32
	Occupational	2 (12.5)	33 (5.3)	.21
	Clustering	1 (6.3)	30 (4.8)	.55
	Contact	4 (25.0)	129 (20.5)	.75
**Pre-existing conditions, n (%)**				
	CAD^c^	2 (0.5)	3 (0.9)	.99
	Diabetes	3 (0.9)	11 (2.5)	.24
	Hypertension	9 (3.3)	19 (4.4)	.67
	COPD^d^	0 (0.0)	3 (0.7)	.56
	Malignancy	8 (2.8)	7 (1.8)	.39
	CKD^e^/ESRD^f^	8 (2.8)	5 (1.5)	.37
**Initial symptoms**				
	Dyspnea, n (%)	53 (18.8)	60 (16.4)	.74
	Cough, n (%)	140 (49.8)	226 (62.4)	.31
	Stuffed nose, n (%)	88 (31.3)	166 (45.8)	.31
	Fever, n (%)	78 (27.7)	134 (37.0)	.37
	Body temperature (°C), mean (SD)	36.9 (0.9)	37.1 (1.7)	.69

^a^ED: emergency department.

^b^TOCC: travel, occupation, contact and cluster.

^c^CAD: coronary artery disease.

^d^COPD: chronic obstructive pulmonary disease.

^e^CKD: chronic kidney disease.

^f^ESRD: end-stage renal disease.

### Survey Times

**Figure 8:**
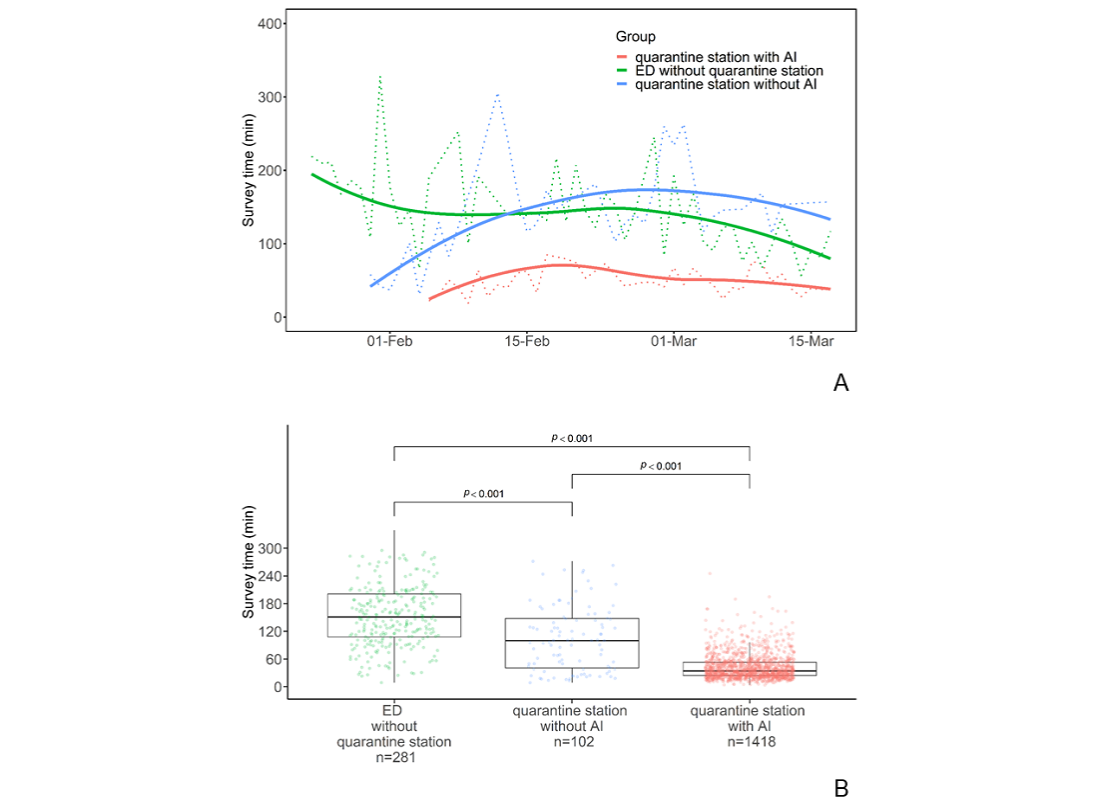
(A) Comparison of survey processing times over the study period between the traditional ED and quarantine station groups with or without AI applications. (B) Box plots of the survey processing times for the traditional ED group, the quarantine station group without AI, and the quarantine station group with AI. AI: artificial intelligence; ED: emergency department; min: minutes.

To further analyze the impact of the AI application on the quarantine station, we subdivided those in the quarantine station into groups with or without AI. As shown in Figure 8, compared with the conventional ED track (n=281), the survey time at the clinical quarantine station (n=1520) was significantly shortened, with a median survey time at the ED of 153.0 minutes (95% CI 108.5-205.0) vs 35 minutes at the clinical quarantine station (95% CI 24-56; *P*<.001). Furthermore, the use of the AI application in the quarantine station reduced the survey time in the station; the median survey time without AI in the quarantine station was 100.5 minutes (95% CI 40.3-152.5), vs 34 minutes with AI in the quarantine station (95% CI 24-53; *P*<.001).

## Discussion

### Principal Findings

In this study, we demonstrate how the NCKUH AI trilogy of diversion to a smart quarantine station incorporated with the SCAS, AI-assisted image interpretation, and a built-in clinical decision-making algorithm improved medical care and reduced the processing time at the quarantine station. In addition, our ex vivo study demonstrated the efficiency of 75% alcohol disinfection of the tablet computer after initial exposure to a positive viral load; by applying this procedure, the tablet computer is rendered safe for use by people in the quarantine station. This enhances patient quality of care and reduces risks to medical staff. Based on Taiwan’s experience with SARS in 2003, the diversion of patients in the ED was crucial for early management and in-hospital infection control. Most Taiwanese hospitals needed to set up clinical quarantine stations near their EDs to survey patients who were suspected of being infected with COVID-19 due to the heavy caseloads experienced by hospitals daily. When a clinical quarantine station is set up, several factors can be detrimental to the conditions in the station. These factors include patient crowding in the restricted space within the quarantine station, high possibility of cross-contamination or coinfection, daily changes and modifications to clinical guidelines by the Taiwan CDC, lack of familiarity of backup physicians with infectious disease control, and scarcity of radiology or pulmonary physicians in most hospitals to provide accurate and immediate pneumonia diagnoses.

To the best of our knowledge, this is the first time a smart quarantine station with a computer featuring a built-in clinical assistance system was used during an epidemic to accelerate complex workflows. Compared to the conventional ED track, the efficiency of the survey of the risk of COVID-19 infection was significantly improved by at least threefold for the NCKUH quarantine station. These results also highlight that the efficiency of the risk survey for COVID-19 infections at clinical quarantine stations is of general interest and helps address an unmet clinical need.

Triage in the ED for pandemic infectious disease screening is particularly challenging. Although telemedicine allows patients and physicians to communicate without direct contact, symptomatic or worried patients still directly visit the ED, and they can be efficiently screened by modern triage [22]. For in-person care, patients with positive signs of high-risk features should be immediately isolated to avert further contact with patients and health care workers. According to Hollander et al [22], tablet computers can be cleaned between patients using well-defined infection control procedures. Furthermore, daily updates of the SCAS system help physicians or surgeons who may not be experts in radiologic interpretation of pneumonia patches or infectious disease to make proper decisions with little preparation before assuming their quarantine station duties.

The COVID-19 pandemic is affecting not only patients but also economies, politics, and daily life worldwide. Better management of future pandemics will reduce these human and economic burdens. All medical science personnel should use lessons learned from past and present pandemics to improve upon past performance to prevent catastrophic effects of future disease outbreaks.

### Conclusions

We demonstrated a feasible, safe, and scientific application of a smart device with a built-in algorithm combined with an AI-based image system in a quarantine station to facilitate the survey process, avoid cross-infection, and reduce the burden on team members, including physicians, nurses, and technicians. This helpful process should be adapted into our strategies to address emerging endemic infectious diseases in the future.

## Abbreviations

AI: artificial intelligence
CDC: Centers for Disease Control
Cp: crossing point
CT: computed tomography
ED: emergency department
GGO: ground-glass opacity
MERS: Middle East respiratory syndrome
NCKUH: National Cheng Kung University Hospital
ReLU: rectified linear unit
RdRp: RNA-dependent RNA polymerase
RT-PCR: real time–polymerase chain reaction
SARS: severe acute respiratory syndrome
SARS-CoV: severe acute respiratory syndrome coronavirus
SCAS: smart clinical assisting system
TOCC: travel, occupation, contact, and cluster
